# School-based exposures to oil and gas development for public school children in the United States

**DOI:** 10.1038/s41370-026-00864-9

**Published:** 2026-03-28

**Authors:** Cassandra J. Clark, Erin Campbell, Stephanie T. Grady, Jonathan Buonocore, Amira Aker, Nicole C. Deziel, Joan A. Casey, Mary Willis

**Affiliations:** 1 Division of Pediatric Epidemiology & Clinical Research, Department of Pediatrics, University of Minnesota School of Medicine, Minneapolis, MN, USA.; 2 Department of Environmental and Occupational Health, George Washington University Milken Institute School of Public Health, Washington, DC, USA.; 3 Department of Epidemiology, Boston University School of Public Health, Boston, MA, USA.; 4 Department of Environmental Health, Boston University School of Public Health, Boston, MA, USA.; 5 Department of Environmental Health Sciences, Yale School of Public Health, New Haven, CT, USA.; 6 Department of Occupational and Environmental Health Sciences, University of Washington School of Public Health, Seattle, WA, USA.; 7 Department of Epidemiology, University of Washington School of Public Health, Seattle, WA, USA.

**Keywords:** Exposure assessment, Environmental disparities, Children’s environmental health, Environmental justice, Oil and gas development

## Abstract

**BACKGROUND:**

Oil and gas development (OGD) can release numerous hazards, such as air and water pollution. Residential proximity to OGD is associated with adverse health outcomes in children, including birth defects and cancer.

**OBJECTIVE:**

While children spend significant time at school, little is known about school-based exposure. We quantified the number of K-12 U.S. schools near OGD and evaluated whether exposure varied by school-level sociodemographic status.

**METHODS:**

We combined public school data from the National Center for Educational Statistics with OGD well location data. We estimated proximity and density of active OGD within 800 m, 1.6 km, and 10 km buffers for each school during the 2022–2023 school year. We used logistic regression with state fixed effects to estimate associations between OGD exposure and schools having >50% students of non-Hispanic White race, Hispanic ethnicity, and free/reduced lunch eligibility, overall and stratified by rurality.

**RESULTS:**

29,649 (29.2%) of U.S. public schools were within 10 km of OGD. Overall, predominantly White (OR: 1.37, 95% CI: [1.32–1.43]) and free/reduced lunch eligibility (1.14 [1.09–1.19]) schools were more likely to be within 10 km of OGD. In rural areas, schools with predominantly Hispanic and free/reduced lunch-eligible students had 1.51 (1.15–1.97) and 1.20 (1.00–1.45) times the odds of being within 800 m of OGD, respectively; this was consistent in micropolitan, but not metropolitan, areas. Schools with predominantly non-Hispanic White students were more likely to be near OGD (800 m: 2.10 [1.93–2.27]) only in metropolitan areas.

**SIGNIFICANCE:**

Over 14.5 million students attended schools within 10 km of OGD in 2022–2023. These schoolchildren often disproportionately came from persistently marginalized groups compared to their less-exposed peers, and patterns varied strongly by urbanicity. Exposure to OGD while at school may harm students’ health and academic development, especially among children in low-resource settings.

**IMPACT STATEMENT:**

This study provides new information on estimating exposure to OGD in U.S. public schools nationwide. More than 14.5 million U.S. public school students were potentially exposed to OGD during the 2022–2023 school year, and these school children tended to be from consistently marginalized groups. Exposure to OGD at school may be detrimental to students’ health and academic development, and these effects may be amplified in low-resource settings. This work has potential health implications for any state with oil and gas development, which should be considered in ongoing policy discussions on public health protection, particularly as regulations change.

## INTRODUCTION

**Fig. 1:**
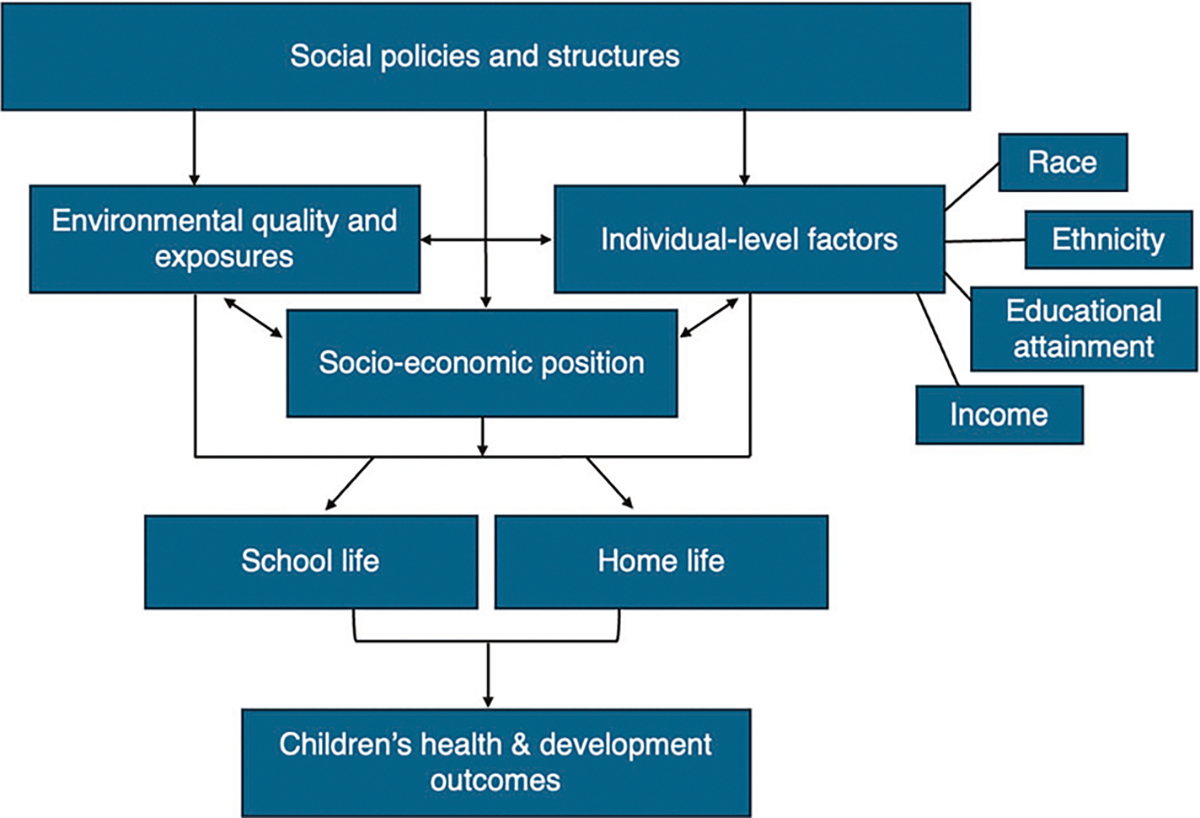
Both the home and school environments that children experience can affect their long-term health and development outcomes. These factors, such as level of exposure to oil and gas development, may vary between the home and school environments.

Oil and gas development (OGD) is a major form of energy production in the United States and worldwide [1]. An estimated 18 million people in the U.S. live near an OGD site (i.e., a well) [2]. Residential proximity to OGD has been associated with numerous adverse health outcomes in both adults and children [3, 4], including reduced birth weight, birth defects, asthma, and childhood cancer [5–13]. In fact, epidemiologic studies to date have generally used residential locations to assign OGD exposure in relation to adverse health outcomes [14]. However, children may spend up to half of their waking hours at school, meaning that the majority of their daily exposures may occur outside of the residence. Little is known about school-based exposures to OGD (Fig. 1).

Children attending school near OGD may be exposed to air and water pollutants, such as particulate matter, endocrine-disrupting chemicals, and indoor radon [14–22]. Elevated noise levels near OGD sites also raise health concerns [23, 24], particularly for children’s sleep quality, mental health, and cardiovascular health [25, 26]. Despite plausible exposure pathways, data on OGD-related exposures in public schools are sparse. However, studies of non-OGD school-based exposures have documented elevated levels of pollutants, such as lead, volatile organic compounds, and fine particulate matter, as well as other health hazards, such as high heat [27–35]. These studies document persistent inequalities in who is exposed to these hazards; for instance, schools with higher proportions of students receiving subsidized lunches were more likely to be located near concentrated animal feeding operations [31]. OGD near schools poses similar risks, potentially affecting student health and academic performance [28, 35].

The effects of school-based exposures to OGD may be amplified among marginalized populations, who may have fewer resources available while bearing a disproportionate burden of exposure (i.e., an environmental injustice) [36–43]. However, existing literature for OGD has focused exclusively on residential exposures. For instance, OGD sites are more likely to be located near homes with a lower market value in Colorado [44] and higher poverty communities in Pennsylvania [45]. OGD waste disposal sites are more likely to be sited in low-income areas in Ohio [46] and communities of color in Texas [47, 48]. Rural communities have been frequently impacted by the expansion of OGD [49], and rurality is an important predictor of OGD siting [50]. Building on this evidence, we hypothesize that children and adolescents in lower-resource communities may also experience disproportionate exposure to OGD at their school location, potentially compounding their residential exposures and associated health risks, and that school rurality may influence these associations [31, 51]. Understanding school-related exposure to OGD is critical to developing health-protective policies.

To quantify potential exposures incurred by children while at school, we performed a cross-sectional national evaluation of the proximity of U.S. K-12 public schools to OGD, focusing on potential sources of environmental injustice.

## METHODS

### Data Sources

Our underlying national-scale exposure data set is from the Oil and Gas Mapping database (OGIM) v2.5.1 (https://zenodo.org/records/13259749) [52]. In states/provinces with high missing rates for temporal data for OGD activity, we supplemented these data with additional publicly available state agency-reported data. Several states (e.g., Arkansas, Illinois, Michigan, South Dakota, Tennessee, Oklahoma, Mississippi) had observations in OGIM that lacked date information. We merged date information onto these observations from publicly available state sources. Idaho, Nebraska, and Nevada had no observations in OGIM. We appended observations with dates to the OGIM data set from state sources [53]. We did not impute missing location or time data; any observation missing latitude, longitude, or a usable date was excluded. This integrated dataset estimates OGD site locations (latitude and longitude), key dates (e.g., spud date, completion date), production type (e.g., oil, gas), and drilling type (e.g., horizontal, directional, vertical).

We obtained U.S. public school data from the National Center for Education Statistics (NCES, https://nces.ed.gov/programs/edge/), an aggregate database from state educational agencies. At the time of writing, more than 100,000 public and public charter schools are captured by this database, representing 82% of elementary and secondary school students (typically aged 6–18 years) in the U.S. Data from Alaska and Hawaii are not included, nor are private schools. These data include geographic location (latitude and longitude), administrative attributes (e.g., school type and level), and demographic characteristics (e.g., race and ethnicity of the student population, student free- and reduced-lunch status). For demographic characteristics, the number of students in each category is reported; the percentage of the total student body population was calculated by dividing the number of students in each category by the total enrollment of the school. A total of 101,390 schools were included in the analysis. *N* = 1671 schools were excluded due to missing student enrollment numbers, leaving a final sample size of *n* = 99,719.

### Exposure assessment

We estimated exposure to active OGD sites during the 2022–2023 school year, anchored on July 1-June 30 to account for varying regional schedules. Due to the complexity of COVID-19, we elected to focus on the most current available data that was likely to be unaffected by the COVID-19 pandemic, which was the 2022–2023 school year. We estimated exposure to OGD based on distance to nearest active well. We elected to focus on distance to the nearest well because it is the most policy-relevant and interpretable measure. Once a site is drilled, we consider that site active until 30 years after earliest date of activity to estimate the producing lifespan of each site. Exposure measures were operationalized at the 800 m, 1.6 km, 10 km buffer distances for the main analysis, as the health-relevant setback distances for OGD are not fully understood [14]. We also generated 1 km and 5 km buffer distances as supplemental analyses that may be useful in certain policy contexts. For each buffer size, schools located outside of the buffer were considered unexposed.

### Assessment of school characteristics

For all public schools, NCES provides data on the number of students by 1) race and ethnicity, and 2) eligibility for free or reduced-cost lunch. We measured socio-economic positioning using these two proxy variables from NCES data. These proxies for socio-economic position enabled us to examine who bears the burden of school-based exposure to OGD, a potential source of environmental injustice. Reported race and ethnicity represent an imperfect proxy for the racialized experience of the student population and exposure to structural racism [54–57]. However, given that we are limited to publicly available school data, it is the best available proxy for structural racism, despite its imperfection in capturing its full meaning. Relatedly, student free and reduced lunch status represented a coarse proxy for low-income background, as to be eligible for free and reduced lunch means to be below the Department of Agriculture’s Income Eligibility Guidelines [58]. For the 2022–2023 school year, free and reduced lunch eligibility was determined based on household income, with students eligible for free lunch if their household income was below 130% of the federal poverty level, and students eligible for reduced lunch if their household income was between 130%–185% below the federal poverty level [59]. We evaluated the characteristics of each school using proportions to determine what share of the school’s student body fell in each category. For the analysis, we operationalized the following groups based on sample size: schools with >50% non-Hispanic White students (dichotomous), >50% Hispanic ethnicity (any race) students (dichotomous) and >50% free or reduced cost lunch students (dichotomous); we acknowledge that these categories encompass a diverse range of racial and ethnic identities. We characterized schools as “predominantly” non-Hispanic White, Hispanic, or Free/Reduced Lunch if 50% or more of the students fell into each respective category. To ensure a sufficient sample size for this analysis, a cutoff of 10% of the student population on average was used to determine whether stratification was possible.

Population density, urbanicity, and rurality are important predictors of where OGD activity is sited [50]. Therefore, we linked each school to their ZIP code-level Rural-Urban Commuting Area (RUCA) [60] code: Metropolitan (1–3), Micropolitan (4–6) and Rural (7–10) to evaluate the potential influence of rurality on school characteristics and exposure to OGD. Due to some ZIP codes having too few commutable addresses, some schools were not assigned a RUCA code (*n* = 159, <0.2% of total schools); these schools were assumed to be Rural.

### Statistical analysis

We described patterns in school-based exposure metrics by race, ethnicity, and free/reduced lunch eligibility. Using bivariable logistic regression with state fixed effects, we estimated the associations (odds ratios [OR] and corresponding 95% confidence intervals [CI]) between OGD exposure metrics (exposure) and each school having >50% of students of non-Hispanic White race, Hispanic ethnicity, and free/reduced lunch eligibility (outcome). We stratified by urbanicity to examine the influence of rurality on the distribution of OGD exposures. In accordance with modern statistical methods, our approach to interpreting data was to evaluate the magnitude, direction, and precision of the effect estimates [61]. Exposure measures were derived using R Statistical Software version 4.2.2., and statistical analyses were conducted in SAS 9.4.

### Sensitivity analyses

As a supplemental sensitivity analysis, we replicated our main model results using County-level fixed effects. Additionally, we also defined “predominant” characteristics of the student body as either 40% and 60% of the student body, respectively. Finally, we conducted a sub-analysis focused only on the top oil and gas-producing states as designated by the U.S. Energy Information Administration: Alaska, Colorado, Louisiana, North Dakota, New Mexico, Pennsylvania, Oklahoma, Texas, and West Virginia.

## RESULTS

### School characteristics

**Fig. 2:**
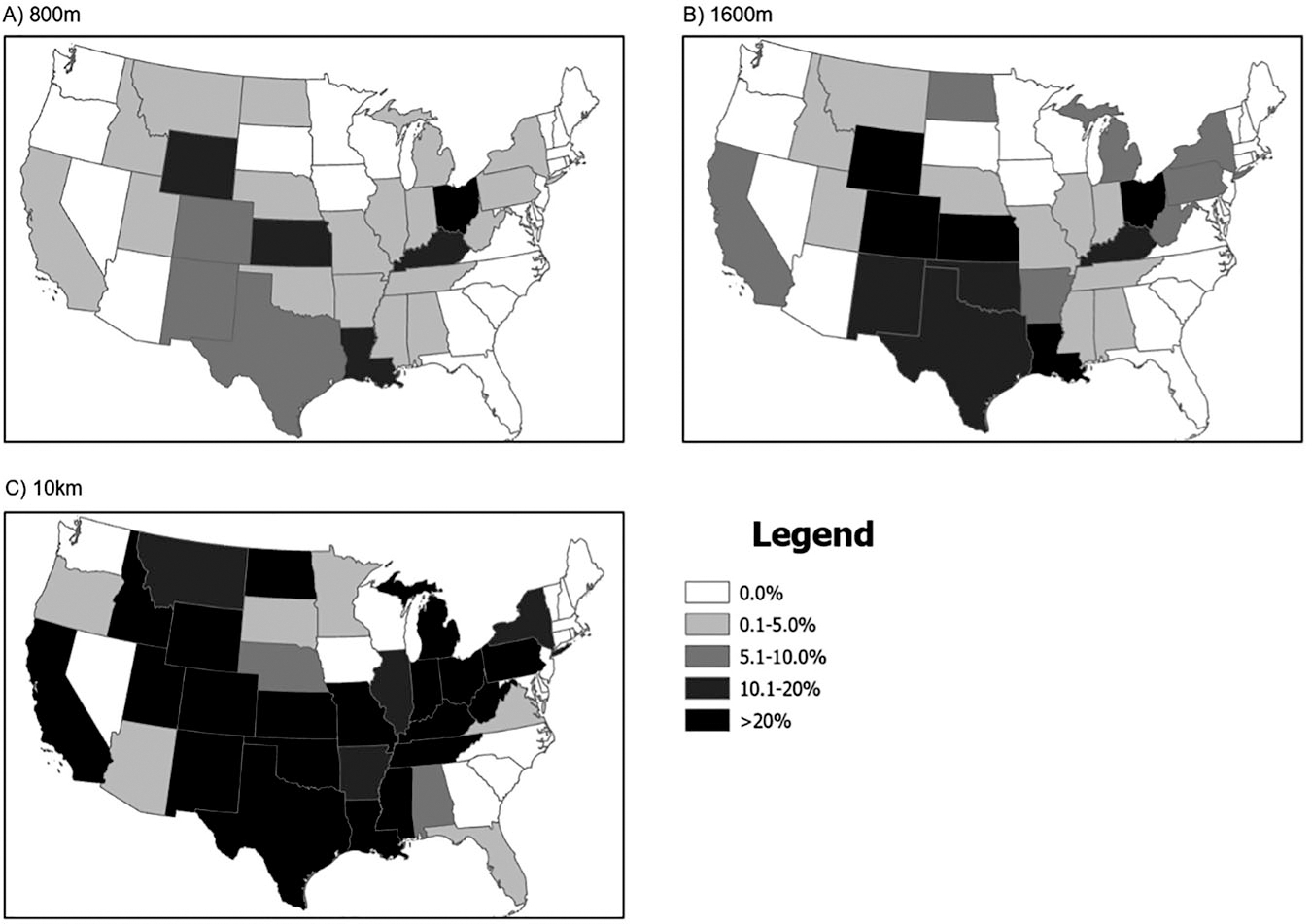
Percent of K-12 public schools exposed to oil and gas development within 800 m, 1.6 km, and 10 km per state. **A** uses an 800 m buffer size; **B** uses a 1.6 km buffer size; **C** uses a 10 km buffer size.

In the 2022–2023 school year, 3,837 (3.8%) of U.S. public schools were within 800 m of active OGD, 7742 (7.6%) were within 1.6 km, and 29,649 (29.2%) were within 10 km (Table 1), representing more than 1.7, 3.6, and 14.5 million schoolchildren, respectively. Schools were exposed to an average of 4.5, 9.0, and 24.0 wells within 800 m, 1.6 km, and 10 km, respectively (Table S1). The states with the greatest proportion of exposed schools (within 1.6 km) included Ohio, Kentucky, Oklahoma, Wyoming, Colorado, Kansas, New Mexico, Texas, and Louisiana (Fig. 2).

### School-level characteristics associated with proximity to oil and gas development

There were several differences in demographic and economic characteristics of schools located near OGD compared to those located farther away (Table 1). Schools within 1.6 km of OGD had higher mean percentages of non-Hispanic White students (57–62%) compared to schools further from OGD (47–48%). The relationships between OGD exposure and percentage of Hispanic students varied, with schools within 800 m of OGD having 21.6% Hispanic student body on average compared to 26.3% in schools further away. There was little difference between the mean percentages of Free/Reduced Lunch students for schools within 1.6 km and 800 m of OGD compared to schools further away (~2%). Conversely, schools within 10 km of OGD had higher mean percentages of Hispanic students (27.9% vs. 25.4%), non-Hispanic White students (50.4% vs. 47.4%), and Free/Reduced Lunch-eligible students (59.1% vs. 58.5%). This relationship remained relatively consistent at 5 km (Table S2).

### School student body socio-demographic characteristics associated with proximity to oil and gas development

We found that predominantly (>50%) non-Hispanic White schools (*n* = 48,449 schools) had greater odds of being located within 800 m, 1.6 km, and 10 km of OGD (Table 2) than schools with <50% White students. Predominantly non-Hispanic White schools had 2.10 times the odds of exposure to OGD within 800 m (95% CI: 1.93–2.27) as compared to other schools. The odds of OGD exposure for predominantly non-Hispanic White schools consistently increased in magnitude as the buffer size grew smaller; at larger buffer sizes, this association was somewhat attenuated (10 km OR: 1.37, 95% CI: 1.32–1.43). Using County-level fixed effects did not result in substantial change (Table S3). These associations varied by state (Table S4). For instance, in Colorado, predominantly non-Hispanic White schools had 0.76 times the odds of being within 1.6 km of OGD (95% CI: 0.60–0.95). Conversely, in Pennsylvania, Oklahoma, Texis, and Louisiana, predominantly non-Hispanic White schools were more likely to be within 1.6 km of OGD than other schools.

In models not adjusting for rurality, predominantly Hispanic schools (*n* = 18,438 schools) were less likely to be near OGD (Table 2) than their counterpart schools at all buffer sizes (Table S5). This pattern also varied by state (Table S4). In Colorado, predominantly Hispanic schools were more likely to be within 1.6 km of OGD (OR: 1.77, 95% CI: 1.39–2.26), though not 10 km (OR: 1.22, 95% CI: 0.85–1.74). Similarly, predominantly Hispanic schools in Texas were more likely to be within 10 km of OGD (OR: 1.23, 95% CI: 1.13–1.34). Conversely, in New Mexico, predominantly Hispanic schools were significantly less likely to be within 1.6 km (OR: 0.21, 95% CI: 0.13–0.35) and 10 km (OR: 0.52, 95% CI: 0.39–0.69) of OGD.

Schools with predominantly free/reduced lunch-eligible students were more likely to be within 10 km of OGD than schools with <50% free/reduced lunch students (OR: 1.14, 95% CI: 1.09–1.19; Table 2). Schools with predominantly free/reduced lunch-eligible students were also more likely to be within 1.6 km of OGD (OR: 1.08, 95% CI: 1.02–1.15) than other schools. These associations were consistent at the 5 km buffer size (Table S5). Sensitivity analyses where the “predominant” student body characteristic was defined as 40% or 60% of the student body yielded similar results for all models (Table S6). Associations between free/reduced lunch-eligible student body and OGD exposure varied by state (Table S4). In Colorado and Texas, schools with predominantly free/reduced-lunch eligible students were more likely to be within 10 km of OGD; in New Mexico, these schools were less likely to be within 10 km of OGD.

### Urbanicity and rurality

Urbanicity was an effect modifier of the relationships between school characteristics and OGD exposure. While predominantly non-Hispanic White schools had much greater odds of being within 800 m and 1.6 km of OGD in Metropolitan areas (70–120%), this association was not present in Micropolitan or Rural areas (Table 3). Conversely, predominantly Hispanic schools in Rural areas were 1.51 (95% CI: 1.15–1.97) and 1.37 (95% CI: 1.10–1.71) times more likely to be within 800 m and 1.6 km of OGD, respectively, and less likely to be near OGD in Metropolitan areas at all buffer sizes (e.g., 800 m OR: 0.71, 95% CI: 0.62–0.80). Predominantly free/reduced lunch schools in Rural areas were 20–34% more likely to be within 800 m, 1.6 km, and 10 km of OGD (Table 3). This pattern was not evident in Micropolitan or Metropolitan areas, though predominantly free/reduced lunch schools in Metropolitan areas did experience a moderate increase in odds of OGD within 10 km (OR: 1.11, 95% CI: 1.06–1.17).

## DISCUSSION

We quantified proximity–based exposure to OGD for nearly 100,000 public schools in the contiguous U.S. Schools with higher proportions of non-Hispanic White students and those eligible for free or reduced lunch were more likely to attend schools near OGD sites. However, these exposure relationships were strongly modified by urbanicity. Predominantly Hispanic schools had a strongly increased likelihood of exposure in rural and micropolitan settings, while predominantly non-Hispanic White schools had an increased likelihood of exposure in metropolitan settings. These associations were also highly variable by state, indicating regional differences. We hypothesize that this school-based exposure disparity could have long-term consequences for the health and well-being of these children.

To our knowledge, this is the first peer-reviewed publication to systematically quantify school-based exposures to OGD at the national level. We found that more than 14 million U.S. public school students attended schools within 10 km of active OGD during 2022–2023. Moreover, these children were more likely to be Hispanic or eligible for the free or reduced lunch program in rural and micropolitan areas or White in metropolitan areas as compared to children from unexposed schools. These results align with existing literature indicating that residential OGD exposure is differentially distributed based on socio-economic and demographic factors [45, 62–65]; for example, both OGD and waste disposal wells are more likely to be sited in rural low-income [44–46] and Hispanic communities [47, 48]. OGD also follows patterns of historical redlining, a racially discriminatory housing, lending, and urban planning practice; for instance, in thirteen states with OGD activity, redlined neighborhoods contained twice as many wells as neighborhoods with higher Home-Owners Loan Corporation grades [65]. In our study, schools with predominantly White students were also more likely to be exposed to OGD, which could reflect literature documenting the high proportions of White individuals living in rural areas [50, 66–68].

Both residential and school proximity to OGD may reflect economic disenfranchisement and environmental injustice. Principally, the economic benefits associated with OGD are generally not experienced at the individual level, but rather at the municipal, regional, and corporate level. This is due to legal arrangements, such as separation of surface and subsurface property and mineral rights. In Colorado, discrepancies between the owners of surface rights and mineral rights for the same land have been observed, with only 36–57% of mineral rights being owned by the surface owner [44]. A similar study in Texas reported that 61% of mineral rights owners lived far from the drilling site(s) that they owned the rights for, meaning that they did not experience the associated environmental burdens [69]. OGD siting is complex and variable, following both geological formations as well as environmental justice patterning.

The growing body of literature on OGD indicates an elevated risk of adverse health effects, including childhood cancer, associated with residential exposures up to 10 km [3–8, 12, 13]. Since children spend a significant amount of time per day in school settings, it is biologically plausible that exposure in these environments could result in similar outcomes, particularly since young children may spend a good portion of their day outside. If the exposures incurred at home and at school are significantly different, using the home location solely to assign exposure without considering school exposures could result in exposure misclassification.

Our findings highlight ongoing environmental justice concerns by examining schools as a second major site, beyond the home, where children have experience significant exposure to OGD. Outside of a news article [70], only one published study to date has directly characterized the environment of schools near OGD, attributing much of the pollution to indoor sources related to the school infrastructure [71]. The broader literature shows that schools that serve predominantly racially, ethnically, and socioeconomically marginalized students are often co-located with highways and industrial sites. For example, 16,979 schools serving 6.4 million students were located within 250 m of a major roadway [72], and schools serving predominantly Black students and students eligible for free or reduced lunch were more likely to be sited in these areas [72]. Similarly, a study by the U.S. Environmental Protection Agency demonstrated that over 40% of public schools are located within 800 m of ten or more pollution sources (e.g., Brownfield or Superfund sites) [73], disproportionately exposing children at special education schools. American Indian or Alaska Native children are more likely to be exposed to wildfire smoke at school [74], and rural Native communities may have different regulatory protections based on federal or state recognition. Redlining, which affects schools as well as residential housing, is one potential factor underscoring these exposure disparities. For instance, schools in historically redlined neighborhoods in New York City experienced smaller decreases in air pollution over time than schools in higher-graded neighborhoods [75]. In combination with existing literature, our results reinforce the need to mitigate exposure for vulnerable student populations.

Based on the epidemiologic and exposure science literature, communities often want to know how to protect themselves from OGD exposure. At the policy level, one of the main public health protections in place is setback distances, a required distance between OGD and sensitive receptor, such as a residence or school. The efficacy of setback distances has been debated by researchers and policymakers, and is an active area of research [76–79]. Current setback distances vary substantially by state, ranging from 150 ft in Ohio to a proposed 3200 ft in California [76]. Colorado has some of the most stringent standards of the drilled states, requiring oil and gas wells be 500 ft from single family homes and 1,00 ft from schools [79]. While a positive step towards exposure prevention, setback distances as a public health protective policy measure have several critical limitations. While recent efforts to extend setback distances for new drilling have yielded positive results in some states, newly enacted setback distances do not apply to wells drilled prior to when the legislation was introduced. The U.S. has been home to decades of OGD activity, and there are thousands of legacy wells across the country that are not subject to the relatively recent introduction of setback legislation. These wells may continue to produce oil, gas, and wastewater for decades and can leak methane and associated hazardous volatile organic compounds even after production is complete [80, 81]. As such, abandoned wells are potentially even more important sources of exposure than newly drilled wells [82]. Additionally, while some states are considering school-specific setbacks (e.g., New Mexico [83]), not every state includes schools in these protections. Finally, setback legislation can be undone at the state level, as was the case in Denton County, Texas. It is critical that researchers and policymakers collaborate to identify new or improve existing policies for protecting the health of communities living and attending school near OGD.

When interpreting the results of this cross-sectional ecologic study, there are several limitations to consider. The exposure surrogates calculated may not represent actual OGD exposure in all cases. Although we combined multiple public data sources to increase the available information about each OGD site, our exposure assessment lacks detailed information about factors, such as production volume, exact lifespan, and flaring, which may lead to exposure misclassification. Further, because state reporting requirements vary significantly [84], we were not able to consistently differentiate between conventional and unconventional drilling. Additionally, our analysis is limited to public schools that report to NCES [85]. Our calculations inherently exclude students who attend private schools or schools on indigenous lands (typically overseen by the Bureau of Indian Education), which are also largely in rural areas and may experience high levels of exposure. We were also limited to the socio-demographic variables included in this dataset, in which were not able to full capture the individual and joint processes resulting from structural racism and the overlap with economic disenfranchisement. Finally, some demographic subgroups had small sample sizes, and we were unable to examine finer categories of race and ethnicity. Nevertheless, our study provides important data on school-based OGD exposures for a majority of public elementary and secondary school students in the U.S.

## CONCLUSION

This study provides new information on estimating exposure to OGD in U.S. public schools nationwide. More than 14.5 million U.S. public school students were potentially exposed to OGD during the 2022–2023 school year, and these school children tended to be from consistently marginalized groups. School-based exposure to OGD may be detrimental to students’ health and academic development, and these effects may be amplified in low-resource settings. This work has potential health implications for any state with OGD activity. Our results should be considered in ongoing policy discussions around public health protection, particularly as regulations change and evolve.

## Supplementary Material

Supplementary Material

**Supplementary information** The online version contains supplementary material available at https://doi.org/10.1038/s41370-026-00864-9.

## Figures and Tables

**Table 1.** Characteristics of schools exposed to oil and gas development vs.

Buffer Size	800 m			1.6 km			10 km			> 10 km		
Characteristic	Exposed schools			Exposed schools			Exposed schools			Unexposed schools		
	Any well within 800 m			Any well within 1.6 km			Any well within 10 km			No wells within 10 km		
Number (%) of schools	3837 (3.8)			7742 (7.6)			29,648 (29.2)			71,742 (70.8)		
Estimated number of students	1,761,632			3,601,011			14,514,481			35,938,652		
Percentage of student body (2022–2023)												
	Mean	Median	(IQR)	Mean	Median	(IQR)	Mean	Median	(IQR)	Mean	Median	(IQR)
Non-Hispanic White	61.9	74.0	(33.3–91.0)	57.4	66.7	(25.5–88.9)	50.4	53.9	(15.9–83.2)	47.4	49.5	(15.3–76.9)
Hispanic	21.6	7.4	(2.5–31.0)	24.9	10.1	(3.1–39.4)	27.9	14.0	(4.2–46.2)	25.4	14.7	(6.0–36.3)
Black	8.5	1.6	(0.4–8.1)	9.4	1.8	(0.5–9.5)	11.7	2.6	(0.7–2.3)	0	4.8	(1.0–9.7)
Asian or Pacific Islander	1.3	0	(0.0–0.4)	1.4	0.1	(0.0–0.4)	1.6	0.2	(0.0–0.5)	0	0.2	(0.0–0.6)
Free or reduced lunch eligible	55.6	55.0	(37.0–55.6)	57.2	57.1	(38.5–77.1)	59.1	59.9	(39.0–81.2)	58.5	56.0	(34.0–80.4)

*IQR* Interquartile range.

Unexposed schools based on 800 m, 1.6 km, and 10 km buffer sizes (total n schools = 99,719).

**Table 2.** Odds of being exposed to oil and gas development for schools with predominantly non-Hispanic White, Hispanic, and Free/Reduced Lunch student bodies as compared to other schools with state fixed effects.

	Any well within 800 m	Any well within 1.6 km	Any well within 10 km
	OR (95% CI)	OR (95% CI)	OR (95% CI)
School type	>50%	>50%	>50%
Predominantly White			
Yes (*n* = 48449)	2.10 (1.93–2.27)	1.75 (1.65–1.86)	1.37 (1.32–1.43)
No (*n* = 51270)	1.00	1.00	1.00
Predominantly Hispanic			
Yes (*n* = 18438)	0.78 (0.70–0.86)	0.90 (0.83–0.96)	0.92 (0.88–0.97)
No (*n* = 81281)	1.00	1.00	1.00
Predominantly Free/Reduced Lunch			
Yes (*n* = 50602)	1.03 (0.96–1.12)	1.08 (1.02–1.15)	1.14 (1.09–1.19)
No (*n* = 49117)	1.00	1.00	1.00

* Predominant: >50% of the student body. *CI* Confidence interval, *OR* Odds ratio. All models are bivariable with state fixed effects.

**Table 3.** Odds of being exposed to oil and gas development for schools with predominantly non-Hispanic White, Hispanic, and Free/Reduced Lunch student bodies as compared to other schools, stratified by urbanicity with state fixed effects.

	Rural (n = 14132)			Micropolitan (n = 11163)			Metropolitan (n = 74424)		
	Any well within 800 m	Any well within 1.6 km	Any well within 10 km	Any well within 800 m	Any well within 1.6 km	Any well within 10 km	Any well within 800 m	Any well within 1.6 km	Any well within 10 km
School type	OR (95% CI)	OR (95% CI)	OR (95% CI)	OR (95% CI)	OR (95% CI)	OR (95% CI)	OR (95% CI)	OR (95% CI)	OR (95% CI)
Predominantly White									
Yes (*n* = 48449)	0.86 (0.70–1.06)	0.94 (0.80–1.11)	1.07 (0.94–1.22)	1.19 (0.93–1.52)	1.04 (0.87–1.24)	1.09 (0.95–1.25)	2.18 (1.97–2.41)	1.73 (1.61–1.87)	1.36 (1.29–1.43)
No (*n* = 51270)	1.00	1.00	1.00	1.00	1.00	1.00	1.00	1.00	1.00
Predominantly Hispanic									
Yes (*n* = 18438)	1.51 (1.15–1.97)	1.37 (1.10–1.71)	1.33 (1.10–1.62)	1.41 (1.02–1.93)	1.49 (1.17–1.89)	1.53 (1.25–1.86)	0.71 (0.62–0.80)	0.88 (0.81–0.95)	0.90 (0.85–0.95)
No (*n* = 81281)	1.00	1.00	1.00	1.00	1.00	1.00	1.00	1.00	1.00
Predominantly Free/Reduced Lunch									
Yes (*n* = 50602)	1.20 (1.00–1.45)	1.29 (1.11–1.50)	1.34 (1.19–1.50)	0.98 (0.80–1.20)	1.18 (1.01–1.38)	1.10 (0.97–1.25)	0.91 (0.82–1.00)	0.96 (0.89–1.03)	1.11 (1.06–1.17)
No (*n* = 49117)	1.00	1.00	1.00	1.00	1.00	1.00	1.00	1.00	1.00

* Predominant: >50% of the student body. CI Confidence interval, OR Odds ratio. All models are bivariable with state fixed effects.

## Data Availability

The datasets used in this study are publicly available from the Oil and Gas Mapping database (OGIM) v2.5.1 (https://zenodo.org/records/13259749) and the National Center for Education Statistics (NCES, https://nces.ed.gov/programs/edge/).
