# Supplementary material for: School-based exposures to oil and gas development for public school children in the United States
Source: J Expo Sci Environ Epidemiol. 2026 Mar 28;36(4):705–12. doi: 10.1038/s41370-026-00864-9 (PMC13112603; doi:10.1038/s41370-026-00864-9)
Supplement: Supplementary file 1 — Supplementary Material [file 41370_2026_864_MOESM1_ESM.docx]

**Supplementary Material for “School-based exposures to oil and gas development for public school children in the United States”**

*Table S1.* *Distance to and number of oil and gas wells within a buffer (based on 800 m, 1.6 km, and 10 km) compared between schools with predominantly non-Hispanic White, Hispanic, and Free/Reduced Lunch student bodies.*

|  | Distance to nearest well (m) | | Number of wells within 800 m | | Number of wells within 1.6 km | | Number of wells within 10 km | |
| --- | --- | --- | --- | --- | --- | --- | --- | --- |
| Predominant* student body characteristic | Mean | Median (IQR) | Mean | Median (IQR) | Mean | Median (IQR) | Mean | Median (IQR) |
| All exposed schools | 3868 | 3382 (1519-5925) | 4.5 | 2 (1-5) | 9.0 | 4 (1-11) | 24.0 | 15 (4-36) |
|  |  |  |  |  |  |  |  |  |
| Non-Hispanic White |  |  |  |  |  |  |  |  |
| Yes (n=48449) | 3728 | 3151 (1264-5895) | 4.8 | 2 (1-5) | 10.2 | 5 (1-13) | 108.7 | 23 (4-127) |
| No (n=51270) | 4006 | 3575 (1778-5952) | 4.0 | 2 (1-4) | 7.3 | 3 (1-8) | 83.4 | 33 (4-104) |
| Hispanic | |  |  |  |  |  |  |  |
| Yes (n=18438) | 3881 | 3371 (1662-5760) | 4.2 | 2 (1-4) | 7.3 | 3 (1-8) | 87.4 | 42 (4-111) |
| No (n=81281) | 3864 | 3385 (1467-5964) | 4.5 | 2 (1-5) | 9.4 | 4 (1-11) | 98.3 | 25 (4-113) |
| Free/Reduced Lunch | |  |  |  |  |  |  |  |
| Yes (n=50602) | 3861 | 3358 (1605-5840) | 4.8 | 2 (1-5) | 8.8 | 4 (1-10) | 95.2 | 37 (5-115) |
| No (n=49117) | 3876 | 3413 (1391-6033) | 4.2 | 2 (1-5) | 9.2 | 4 (1-11) | 96.7 | 18 (3-109) |

**Predominantly: >50% of the student body; IQR: Interquartile range.*

*Table S2. Characteristics of schools exposed to oil and gas development vs. unexposed schools based on 1 and 5 km buffer sizes.*

| Buffer Size | 1 km | | | | | | 5 km | | | | | |
| --- | --- | --- | --- | --- | --- | --- | --- | --- | --- | --- | --- | --- |
| Characteristic | Exposed schools | | | Unexposed schools | | | Exposed schools | | | Unexposed schools | | |
|  | (<1 km from OGD) | | | (>1 km from OGD) | | | (<5 km from OGD) | | | (>5 km from OGD) | | |
| Number (%) of schools | 4904 (4.8) | | | 96486 (95.2) | | | 19807 (19.5) | | | 81583 (80.5) | | |
| Estimated number of students | 2258729 | | | 48193599 | | | 9654829 | | | 40798008 | | |
|  |  |  |  |  |  |  |  |  |  |  |  |  |
| Percentage of student body (2022-2023) | |  |  |  |  |  |  |  |  |  |  |  |
|  | Mean | Median | (IQR) | Mean | Median | (IQR) | Mean | Median | (IQR) | Mean | Median | (IQR) |
| Non-Hispanic White | 60.6 | 71.8 | (30.9-90.5) | 47.6 | 49.8 | (14.9-77.8) | 51.2 | 55.5 | (16.2-84.3) | 47.5 | 49.8 | (15.3-77.3) |
| Hispanic | 22.6 | 8.1 | (2.6-34.1) | 26.3 | 14.8 | (5.7-39.1) | 27.9 | 13.6 | (4.0-46.4) | 25.7 | 14.7 | (5.9-37.4) |
| Non-Hispanic Black | 8.7 | 1.6 | (0.4-8.3) | 14.8 | 4.2 | (0.9-17.9) | 11.4 | 2.5 | (0.6-12.1) | 15.2 | 4.5 | (1.0-18.8) |
| Non-Hispanic AAPI | 1.4 | 0.0 | (0.0-0.4) | 1.8 | 0.2 | (0.0-0.6) | 1.6 | 0.1 | (0.0-0.5) | 1.8 | 0.2 | (0.0-0.6) |
| Free or reduced lunch eligible | 55.9 | 55.4 | (37.4-75.3) | 57.3 | 57.5 | (35.3-81.0) | 59.1 | 60.0 | (39.5-80.6) | 56.8 | 56.6 | (34.5-80.7) |

*IQR: Interquartile range.*

*Table S3.* *Odds of being exposed to oil and gas development for schools with predominantly non-Hispanic White, Hispanic, and Free/Reduced Lunch student bodies as compared to other schools with County fixed effects.*

|  | Any well within 800 m | Any well within 1.6 km | Any well within 10 km |
| --- | --- | --- | --- |
|  | OR (95% CI) | OR (95% CI) | OR (95% CI) |
| School type | >50% | >50% | >50% |
| Predominantly White | |  |  |
| Yes (n=48449) | 1.86 (1.67-2.06) | 1.61 (1.49-1.74) | 1.28 (1.21-1.35) |
| No (n=51270) | 1.00 | 1.00 | 1.00 |
| Predominantly Hispanic | |  |  |
| Yes (n=18438) | 0.88 (0.76-1.00) | 1.05 (0.96-1.16) | 1.10 (1.03-1.19) |
| No (n=81281) | 1.00 | 1.00 | 1.00 |
| Predominantly Free/Reduced Lunch | |  |  |
| Yes (n=50602) | 0.84 (0.77-0.92) | 0.89 (0.84-0.95) | 0.92 (0.88-0.96) |
| No (n=49117) | 1.00 | 1.00 | 1.00 |

**Predominant: >50% of the student body. CI: Confidence interval; OR: Odds ratio. All models are bivariable and have County fixed effects.*

*Table S4. State-specific odds of being exposed to oil and gas development for schools with predominantly non-Hispanic White, Hispanic, and Free/Reduced Lunch student bodies as compared to other schools among top oil and gas-producing states, designated by the U.S. Energy Information Administration.*

|  | | | | | | |
| --- | --- | --- | --- | --- | --- | --- |
|  | Any OGD well within 1.6 km | | | Any OGD well within 10 km | | |
| Predominant student body characteristic | Non-Hispanic White | Hispanic | Free/Reduced Lunch | Non-Hispanic White | Hispanic | Free/Reduced Lunch |
| State | OR (95% CI) | | | OR (95% CI) | | |
| Top oil and gas-producing states | | | | | | |
| AK* | NA | NA | NA | NA | NA | NA |
| CO | **0.76 (0.60-0.95)** | **1.77 (1.39-2.26)** | 1.00 (0.80-1.27) | 0.72 (0.52-1.00) | 1.05 (0.74-1.49) | **2.17 (1.54-3.05)** |
| LA | **1.41 (1.10-1.81)** | 0.43 (0.15-1.23) | 1.00 (0.73-1.39) | **0.71 (0.53-0.94)** | NA | 1.22 (0.85-1.74) |
| ND | NA | NA | **0.13 (0.02-0.99)** | 0.58 (0.33-1.00) | NA | 0.83 (0.49-1.38) |
| NM | **0.29 (0.09-0.94)** | **0.21 (0.13-0.35)** | 1.12 (0.64-1.96) | 0.64 (0.38-1.08) | **0.52 (0.39-0.69)** | **0.52 (0.38-0.71)** |
| PA | **3.24 (2.13-4.94)** | NA | **1.38 (1.06-1.79)** | **2.46 (1.97-3.06)** | NA | 0.99 (0.84-1.17) |
| OK | **1.44 (1.05-1.96)** | 1.19 (0.68-2.08) | 0.91 (0.64-1.29) | **2.04 (1.63-2.55)** | 1.69 (0.97-2.95) | **0.46 (0.34-0.62)** |
| TX | **1.27 (1.12-1.44)** | 1.00 (0.91-1.12) | 0.96 (0.85-1.07) | **1.27 (1.14-1.41)** | **1.23 (1.13-1.34)** | **1.54 (1.41-1.68)** |
| WV | 1.19 (0.36-3.99) | NA | NA | 0.89 (0.45-1.76) | NA | NA |
| Models adjusted for rurality. NA: Insufficient sample size for model convergence. *Alaska (AK), among the top oil-producing states, had insufficient exposed schools to assess. Models adjusted for rurality. | | | | | | |

*Table S5. Comparison of oil and gas development exposure (1 and 5 km) between schools with predominantly non-Hispanic White, Hispanic, non-Hispanic Black, and Free/Reduced Lunch student bodies.*

|  | Number of wells within 1 km | | Odds of any well within 1 km | Number of wells within 5 km | | Odds of any well within 5 km |
| --- | --- | --- | --- | --- | --- | --- |
| Predominant* student body characteristic | Mean | Median (IQR) |  | Mean | Median (IQR) | OR (95% CI) |
| All exposed schools | 5.6 | 3 (1-6) | - | 18.4 | 10 (2-25) | - |
|  |  |  |  |  |  |  |
| Non-Hispanic White* | |  |  |  |  |  |
| Yes (n=48449) | 6.1 | 3 (1-7) | 2.03 (1.89-2.18) | 18.5 | 10 (2-25) | 1.38 (1.32-1.44) |
| No (n=51270) | 5.0 | 2 (1-5) | 1.00 | 18.4 | 10 (2-25) | 1.00 |
| Hispanic | |  |  |  |  |  |
| Yes (n=18438) | 5.2 | 2 (1-5) | 0.80 (0.73-0.88) | 19.2 | 11 (2-26) | 0.94 (0.90-0.99) |
| No (n=81281) | 5.7 | 3 (1-7) | 1.00 | 18.2 | 10 (2-25) | 1.00 |
| Free/Reduced Lunch | |  |  |  |  |  |
| Yes (n=50602) | 6.0 | 3 (1-6) | 1.03 (0.96-1.11) | 19.0 | 11 (3-26) | 1.15 (1.10-1.20) |
| No (n=49117) | 5.4 | 3 (1-6) | 1.00 | 17.7 | 9 (2-24) | 1.00 |

**Predominant: >50% of the student body; IQR: Interquartile range; CI: Confidence interval; OR: Odds ratio. All models are bivariable and have state fixed effects.*

*Table S6. Sensitivity analysis including 40% and 60% proportions for odds of being exposed to oil and gas development for schools with predominantly non-Hispanic White, Hispanic, and Free/Reduced Lunch student bodies.*

|  | OR (any well within 800 m) | | OR (any well within 1.6 km) | | OR (any well within 10 km) | |
| --- | --- | --- | --- | --- | --- | --- |
| Percent of group threshold | >40% | >60% | >40% | >60% | >40% | >60% |
| >#% Non-Hispanic White |  |  |  |  |  |  |
| Y | 1.92 (1.77-2.08) | 2.32 (2.14-2.51) | 1.65 (1.56-1.75) | 1.98 (1.86-2.10) | 1.26 (1.21-1.32) | 1.54 (1.47-1.61) |
| N | 1.00 | 1.00 | 1.00 | 1.00 | 1.00 | 1.00 |
| >#% Hispanic |  |  |  |  |  |  |
| Y | 0.67 (0.61-0.74) | 0.77 (0.69-0.86) | 0.82 (0.76-0.88) | 0.90 (0.84-0.97) | 0.88 (0.84-0.92) | 0.99 (0.94-1.04) |
| N | 1.00 | 1.00 | 1.00 | 1.00 | 1.00 | 1.00 |
| >#% Free/Reduced Lunch | |  |  |  |  |  |
| Y | 1.20 (1.10-1.30) | 0.91 (0.84-0.99) | 1.23 (1.15-1.30) | 0.97 (0.92-1.03) | 1.20 (1.15-1.25) | 1.08 (1.04-1.20) |
| N | 1.00 | 1.00 | 1.00 | 1.00 | 1.00 | 1.00 |

*CI: Confidence interval; OR: Odds ratio. All models are bivariable and have state fixed effects.*
